# Earthquake in Western Iran: Renovation Kills

**DOI:** 10.1371/currents.dis.ac10620db4a0c944e605c4c226a75f64

**Published:** 2018-08-16

**Authors:** Abbas Ostadtaghizadeh, Mona Khaleghy Rad, Hamidreza Aghababaeian, Mehdi Zare, Farnaz Kamranzad

**Affiliations:** Tehran University of Medical Sciences; Institute for Environmental Research, Tehran University of Medical SciencesTehran University of Medical Sciences; 1 Department of Disaster Public Health, School of Public Health, Tehran University of Medical Sciences, Tehran, Iran. 2 Dezful University of Medical Sciences, Dezful, Iran. 3 Department of Climate Change and Health, Institute for Environmental Research (IER), Tehran University of Medical Sciences, Tehran, IranDepartment of Disaster Public Health, School of Public Health, Tehran University of Medical Sciences, Tehran, Iran; Dezful University of Medical Sciences, Dezful, Iran; International Institute of Earthquake Engineering and Seismology; College of Engineering, University of Tehran, Tehran, Iran

## Abstract

Introduction: Earthquake is the most important cause of death from natural disasters in Iran. This paper brings attention to the main causes of loss of life due to the Kermanshah province earthquake (Nov 12 2017), and provides a wakeup call about the unsafe nature of buildings there. Methods: This study is based on official reports review and a field assessment in the areas affected by the earthquake in western Iran. Results: Although buildings in this area are mainly old structures, strangely, more than 70% of the destroyed buildings in this earthquake were under 5 years of age, newly built or renovated buildings according to mandated building codes. Discussion: Mandated building codes and construction rules and regulations are not respected even for the newly constructed or reconstructed structures buildings. Keywords: Earthquake, Iran, construct, reconstruct, Building codes

## BRIEF INCIDENT REPORT

At 21:48 local time, on Nov. 12, 2017, western Kermanshah Province in Iran was shaken by an earthquake of moment magnitude Mw7.3 for about 30 s^1^ . The epicenter has been reported at the 34.88°N and 45.84°E coordination near the Iran–Iraq border and the depth at 23 km. Based on the active fault map of Iran, this earthquake can be triggered by the movement of the Zagros Mountain Front Fault in Pol-e-Zahab Region1^1^ . In addition, more than 500 aftershocks, greatest of which with magnitude of 4.9 have been recorded by the Iranian Seismological Center.

Due to this earthquake, 620 people died^2^ , 8,000 injured, 70,000 displaced, and over 12,000 buildings damaged^1^ . The total exposed population was 4,700,000 including 75% urban and 25% rural^3^^,^^4^ . Most of the fatalities are reported in Sarpol-e Zahab, Qasr-e-Shirin, and Thalath Babajani. The highest number (559 death) was in the city of Sarpol-e Zahab^2^ around 200 of which from a small area called Fouladi.

Most of the local buildings were one-story adobe, covered with heavy flat roofs. Nevertheless, in larger villages and cities, more-storey brick-based and cement-based buildings were seen. Many of which were newly constructed or reconstructed structures that built according to developed and mandated codes after the bam earthquake of 25 Dec 2003 in Iran. However, around 80% of destructions occurred in buildings with no frame, both in the urban and rural areas^4^ , around 70% of which are visually estimated as buildings of less than 5 years of age, among them two newly constructed hospitals.

Although buildings with no frame in the rural areas of the Kermanshah Province are 15% more than urban areas, number of residents at the buildings with no frame are 60% more in the urban areas than rural^3^ . In addition, from the initial assessments, major fatalities are from the newly constructed or reconstructed structures buildings. Therefore, it is more important than ever to ask, in spite of the implemented building codes, why would newly constructed or reconstructed buildings be destroyed and cause devastation and fatalities of this magnitude.

Indeed, further evaluation of this analysis is required; however, the large number of fatalities, injuries, and displacements due to this earthquake is a wakeup call to the unsafe constructed or reconstructed. Poorly constructed or reconstructed buildings in this area can be due to several factors, including lack of quality control on the architectural and structural designs, owners’ employment of non-expert workers and use of non-standard materials, vaguely defined construction regulations, and owners’ right to hire architects, structural engineers, supervisors and contractors.

Due to insufficient payments to supervisors and lack of restriction of law enforcement on them, surveillance is generally weak in structural designs. On the other hand, architectures are under surveillance of both owners and the city. Monetary deals between owners and supervisors are leading to unsafe structural designs. Law enforcement is another challenge in construction since duties of the institutes are not carefully distributed in the written laws and regulations. Considering these preventive factors, we suggest that some changes in the construction surveillance system and the owners’ rights are required for future building code enforcements.

## CORRESPONDING AUTHOR

Mehdi Zare, Professor of Engineering Seismology, International Institute of Earthquake Engineering and Seismology (IIEES), and Associate member of Geology Division, Department of Basic Sciences; Academy of Sciences of the I.R. Iran. E-mail: mzare@iiees.ac.ir

## COMPETING INTERESTS

The authors have declared that no competing interests exist.

## Data Availability

The data underlying this study have been uploaded to figshare and are accessible using the following DOI: 10.6084/m9.figshare.6949988
